# A validated LC–MS/MS method for analysis of Cabergoline in human plasma with its implementation in a bioequivalent study: investigation of method greenness

**DOI:** 10.1186/s13065-022-00862-6

**Published:** 2022-09-24

**Authors:** Khaled Shalaby, Saleh Alghamdi, Mohammed Gamal, Lobna Mohammed Abd Elhalim, Rehab Moussa Tony

**Affiliations:** 1 Department of Pharmaceutics, College of Pharmacy, Jouf University, P.O. Box 2014, Sakaka, Saudi Arabia; 2 Department of Clinical Pharmacy, Faculty of Clinical Pharmacy, Al Baha University, Al Baha, Saudi Arabia; 3 Pharmaceutical Analytical Chemistry Department, Faculty of Pharmacy, Beni-Suef University, Alshaheed Shehata Ahmed Hegazy St., Beni-Suef, 62574 Egypt; 4 Pharmaceutical Analytical Chemistry Department, Central Administration of Drug Control, Egyptian Drug Authority, Agouza, Giza, 12311 Egypt; 5 Pharmaceutical Analytical Chemistry Department, Faculty of Pharmacy, Modern University for Technology and Information, Cairo, Egypt

**Keywords:** LC–MS/MS, Bioequivalence, Pharmacokinetic, Cabergoline, Dostinex, Bioanalytical method validation

## Abstract

Cabergoline (CAB) is effective prolactin lowering drug. Evaluation of the bioequivalence for the new test product (0.5 mg CAB film-coated tablets) in Egypt is strongly needed for approval of the drug by the official health authority. Therefore, a highly sensitive and rapid (LC–MS/MS) method was validated for CAB analysis in human plasma. CAB was extracted from plasma via diethyl ether using Quetiapine (QUE) as an internal standard. Multiple reaction monitoring (MRM) in positive ion mode was used, *m/z* 452.3 → 381.2 for CAB and 384.2 → 253.1 for QUE. Separation was accomplished on a reversed-phase C_18_. FDA procedures for the bio-analytical method were followed. The method was used in the bioequivalence study to compare the test product (0.5 mg CAB) versus Dostinex tablets, on 24 healthy Egyptian volunteers. The total analysis time was 5.5 min for each sample which permits analysis of various samples per day. The linearity range was from 2.00 to 200.00 pg/mL for CAB. LOD and LOQ were found to be 0.5 and 1.6 pg/mL, respectively. The final greenness numerical value was 0.63 using AGREE tool. The results of pharmacokinetic parameter T_max_ were 2.17, and 2.33 h; for test and reference products, respectively. The generic formulation of test product is considered bioequivalent to the reference product Dostinex 0.5 mg tablets and satisfies the requirements of the Egyptian market. The merits of the method over the previous published methods are low cost; availability of cheap internal standard; rapidness; use of acetonitrile-free solvents mobile phase.

**Supplementary Information:**

The online version contains supplementary material available at 10.1186/s13065-022-00862-6.

## Introduction

**Fig. 1:**
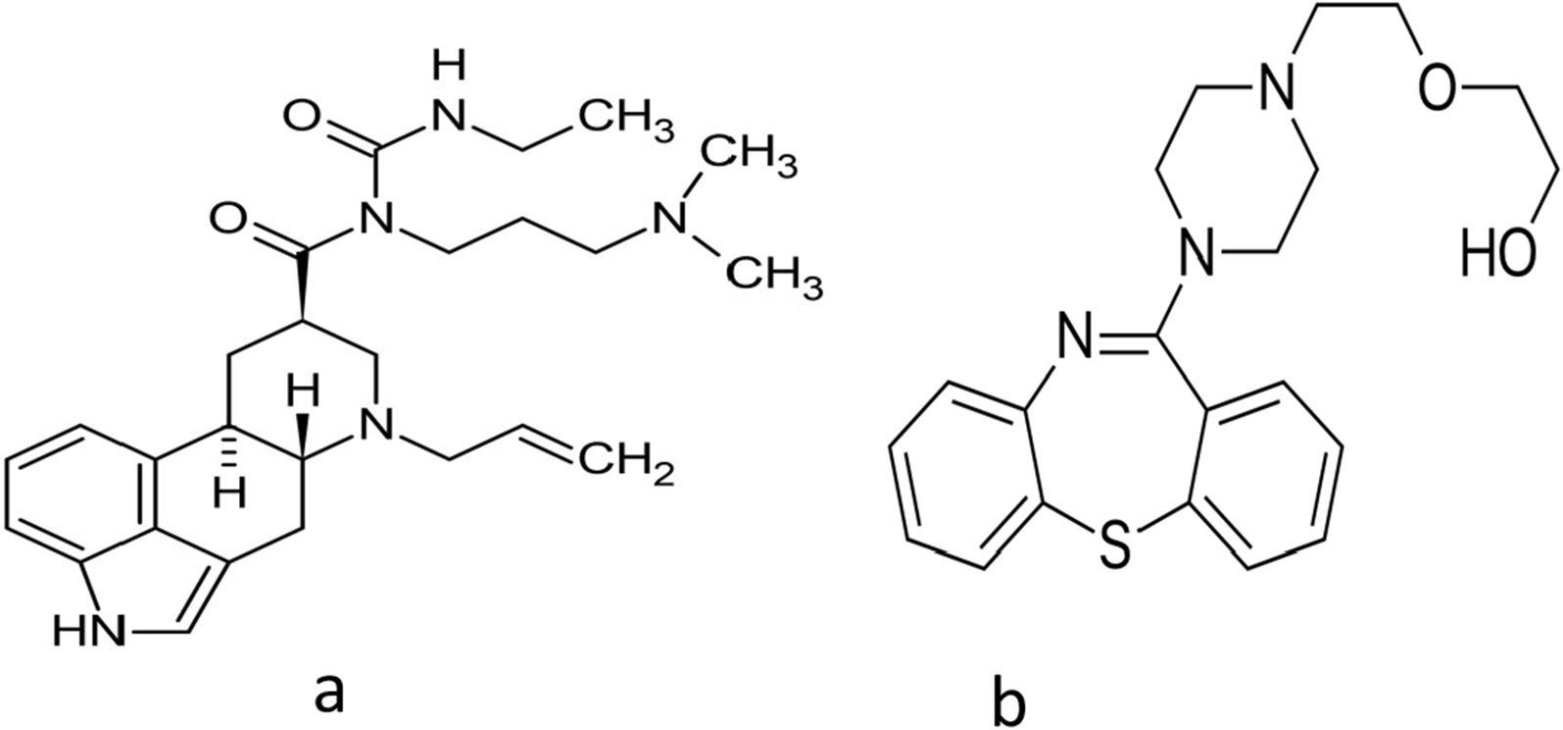
Chemical structure of CAB (**a**) and QUE (internal standard) (**b**)

Cabergoline (CAB) is a potent ergot alkaloid derivative with a long-acting restrained action on prolactin plasma concentration via binding to dopamine receptors in the pituitary gland [1]. Generally, CAB is favored that bromocriptine in terms of tolerability and effectiveness in the treatment of hyperprolactinemia [1]. Besides, it is used for the management of Parkinsonism disease associated with prolactin disorders [2]. The molecular weight of CAB is 451.6 g/mol and its molecular formula is C_26_H_37_N_5_O_2_. The chemical structure of CAB is illustrated in Fig. 1 [3]. Quetiapine (QUE, 383.5 g/mol, C_21_H_25_N_3_O_2_S) was used as an internal standard and its structure is demonstrated in Fig. 1.

CAB is available in Egyptian pharmacies as Dostinex 0.5 mg CAB tablets a brand product of Pfizer, Egypt under license from Parke-Davis Germany, a company of Pfizer INC., USA. Furthermore, other generic formulations are similarly available e.g. Cabergamoun, Marvigoline, Elona, and Nostifix. In the present study, a new formulation will be investigated and compared to the brand product.

Coupling MS with LC and GC greatly improves the selectivity and sensitivity of chromatographic methods [4, 5]. Numerous methods have been recorded for estimating CAB, alone or in combination with other drugs, which includes spectrophotometry [6–8], HPLC [8–12], TLC [10], voltammetry [13–16], and LC/MS/MS [17, 18]. The first LC/MS/MS method [17] which was developed by Allievi and Dostert involved using an expensive Deutrated internal standard which is not available in most QC labs. While the second LC/MS/MS [18] reported by Igarashi et al. was applied for the simultaneous analysis of CAB and L-dopa in human plasma which is suitable for concurrent administration of the two drugs in case of parkinsonism. Besides, large volumes of acetonitrile up to 80% in gradient mode were used in this method, which is not favored in terms of green analytical chemistry. Therefore, there is a need to develop a simple, reliable, green, precise LC/MS/MS method for routine analysis of CAB and performing bioequivalence studies. Many greenness assessment tools were recently reported to evaluate analytical methods qualitatively and quantitatively [19–22]. AGREE approach [21] is the simplest automated dependable software. The greenness score specifies the hazards of analytical methods for analysts and the environment.

Many bioequivalence studies were recorded for CAB including estimation of the effect of grapefruit juice, food, and clarithromycin on the bioavailability of CAB [23–25]. However, no bioequivalence studies were recorded for comparing any generic formulations for CAB and the brand product Dostinex tablets till now. Hence, the main aim of this work was to present a simple, reliable, green, low-cost LC/MS/MS method for routine analysis of CAB using QUE as an internal standard for the first time. Besides, the aim was extended to apply the new LC/MS/MS method in the comparative bioequivalence study to compare test product (0.5 mg CAB film-coated tablets) versus Dostinex 0.5 mg CAB tablets (reference product) a product of Pfizer, Egypt under license from Parke-Davis Germany, a company of Pfizer INC., USA, on healthy Egyptian volunteers under feeding state. This study is the first bioequivalence study for CAB conducted for Egyptian volunteers.

## Experimental

### Materials and reagents

#### Pure drug and internal standard samples

CAB of purity 101.6% was produced by Enaltec labs private limited (Maharashtra, India). The Quetiapine, internal standard, of purity, 99.98% was manufactured by Kopran research laboratory limited (Maharashtra, India).

#### Pharmaceutical products

Test product (0.5 mg CAB film-coated tablets)—Egypt, the product is still investigational and Dostinex 0.5 mg CAB tablets (reference product) a product of Pfizer, Egypt under license from Parke-Davis Germany, a company of Pfizer INC., USA, were purchased from community pharmacy, Cairo, Egypt.

#### Solvents

Formic acid HPLC grade (a minimum of 99.0% purity) was gotten from Dikma Technologies Inc. (CA, USA). Methanol HPLC grade and Water LC/MS grade were got from Merck KGaA (Darmstadt, Germany). All other solvents e.g. acetonitrile, tertiary- butyl methyl ether, ethyl acetate ester, di-chloromethane, and di-ethyl ether were of HPLC grade and were purchased from Sigma Aldrich (Steinheim, Germany). Blank human plasma was friendly obtained from VACSERA (Giza, Egypt).

### Instruments

Chromatographic trails were done via Agilent 6470 LC/TQ triple quadrupole mass system with AJS (Agilent Jet Stream) high-sensitivity ion source Model (G6470A) serial No. SG2018G111, Singapore. The HPLC instrument was composed of a G7167-60101 autosampler, an LC-G7111A pump, and a G7129A column oven (Agilent Technologies Hewlett-Packarrd-Strasse 8763377 Waldbronn, Germany). Balance RADWAG-639514. Multi-tube vortex 060613009 and Cooling Centrifuge HANIL HICRB420032504004 were used.

### LC/MS/MS conditions

A stable isotope-labeled internal standard is usually required to perform MS analysis, whenever available. Though, if the labeled isotope is of high price or not present, a compound with relatively comparable physical characters (pKa and Log P) could be used. Quetiapine (QUE) was selected as a structure correlated compound that is expected to show the same behavior as the investigated drug in both extraction and separation processes.

The mobile phase consisted of 20 mm ammonium acetate and Methanol in the ratio (30: 70, v/v) in isocratic elution mode. Separations were carried out using Agilent eclipse plus C_18_ (100 * 4.6 mm 3.5 µm) column. The flow rate was 0.75 mL/min and the total run time was 5.5 min. The column oven was maintained at 30 °C and the auto-sampler needle rinsing solution was methanol: water (50:50, v/v). The injected volume was 15 µL for each sample.

The mass spectrometer instrument was adapted in positive ion mode with multiple reactions monitoring (MRM). The Agilent 6470 LC/TQ system with AJS (Agilent Jet Stream) high-sensitivity ion source was set up with the following optimized conditions for the target analyte: Gas Temp (300 °C), Gas Flow (9 L/min), Nebulizer gas was adjusted on (20 psi), Sheath Gas Temp (250 °C), and Sheath Gas Flow (8 L/min). Capillary voltage (3000 V), Nozzle Voltage/Charging (0 V). The details of MS conditions are displayed in Table 1.

**Table 1:** LC–MS/MS parameters used for the estimation of CAB and QUE (IS) in human plasma

Analyte	Q1 (m/z)	Q3 (m/z)	D_well_ (s)	CE (V)	Ion mode	Fragmentor voltage (V)
CAB	452.3	381.2	0.2	25	ESI+	135
QUE (IS)	384.2	253.1	0.2	25	ESI+	150

Capillary voltage 3000

*Q1* precursor ion, *Q3* product ion, *CE* collision energy

### Procedures

#### Preparation of stock and working standard liquid solutions

Preparation of stock solutions of (1000.00 µg/mL) for CAB, and (200.00 µg/mL) for QUE was carried out independently in methyl alcohol and stored at 2–8 °C. Suitable dilutions from the stock solutions with methyl alcohol were used for preparation of the working standard solutions (A) of (200.00 ng/mL) for both CAB, and QUE separately. Then further dilutions from the working standard solutions (A) were used to prepare the final working standard solutions of (4.00 ng/mL) and (125.00 pg/mL) for CAB, and QUE respectively using mobile phase as a solvent.

#### Assessment of calibration curves, QC, and plasma samples

Sequential dilutions were performed from the stock solution (4.00 ng/mL) of CAB to prepare 0.02, 0.15, 0.30, 0.50, 1.00, 1.50, 1.80, and 2.00 ng/mL working solutions for calibration standards. Dilutions were done via the mobile phase. Similarly, successive dilutions were done from the stock solution (2 ng/mL) to get 0.06, 0.30, 0.60, and 1.60 ng/mL working solutions for QC samples. Furthermore, 50.00 µL of each working standard was spiked into 450.00 µL blank plasma, then vortex for 1 min to prepare calibration points with concentrations (2.00, 15.00, 30.00, 50.00, 100.00, 150.00, 180.00, and 200.00 pg/mL) standard for the calibration curve and quality control samples with concentrations of 6.00 pg/mL (Low QC), 20.00 pg/mL (medium 1 QC), 60.00 pg/mL (Medium 2 QC) and 160.00 pg/mL (High QC), then all plasma samples were kept at − 70 °C ± 15. After that, sample extraction processes were applied as follows.

#### Sample extraction

Different extracting solvents were tested including Diethyl ether, tertiary-butyl methyl ether, di-chloromethane, ethyl acetate ester, and methanol where diethyl ether was selected due to its highest recoveries percentages. Details of the extraction process were reported as follows, a volume of 50.00 µL from QUE solution (125.00 pg/mL) was spiked into 500.00 µL of Plasma samples, vortex for 1 min, followed by addition of 3.50 mL of Diethyl ether. The samples were vortex-mixed for 3 min and centrifuged at 3500 rpm for 5 min at 5 °C. Then, 3.00 mL of the upper supernatant layer was transferred to Wassermann tubes and evaporated by solvent concentrator at 37 °C, 700 rpm under vacuum. The concentrated sample was reconstituted by 200.00 µL of the mobile phase. Latterly, a volume of 15.00 μL from clear supernatant was injected into LC–MS/MS instrument.

### Method validation

The protocol of the FDA Guidance for Industry Bioanalytical Validation [26] was followed in the validation of the new LC/MS/MS method.

#### Selectivity

The selectivity of the investigated method was evaluated using nine diverse sets of blank human plasma to ensure that the LLOQ response is at least 5 times the blank at the retention time of CAB. The blank plasma samples were estimated as stated before to claim that endogenous plasma components have no chromatographic interference.

#### Carry over

During validation carryover was assessed by injecting blank samples after a high concentration sample or calibration standard at the upper limit of quantification (ULOQ). Carry over in the blank sample following the high concentration standard should not greater than 20% of the lower limit of quantification (LLOQ) and 5% for the IS.

#### Linearity and calibration range

The calibration curve was performed with blank plasma samples (plasma without CAB or QUE), a zero sample (plasma without CAB but with QUE), and non-zero samples (plasma with both CAB and QUE) in the range from 2.00 to 200.00 pg/mL for CAB. The linearity had been evaluated where the ratios of peak areas for CAB and QUE were plotted against the equivalent concentrations. The correlation coefficient (r) should be higher than 0.99 for a good regression equation.

#### Accuracy and precision

Accuracy was assessed on spiked human samples with known concentrations of CAB in five concentration levels [LLOQ, 6.00 pg/mL (Low QC), 20.00 pg/mL (Medium A QC), 60.00 pg/mL (Medium B QC), and 160.00 pg/mL (High QC)]. The QC samples were spiked independently from the calibration standards, using separately prepared stock solutions, unless the nominal concentration(s) of the stock solutions have been established. The assessment of accuracy was expressed as (Recovery, %).

Precision was estimated via the coefficient of variation (CV) for the response area ratio. Precision was demonstrated for the same five concentration levels mentioned in the accuracy. Besides, precision was evaluated by the (RSD, %). To fulfill the acceptance criteria, the variation should be within 15% for all QC samples and not more than 20% for LLOQ.

#### Recovery percent of extracted drug

The recovery of CAB was calculated via comparison of the extracted samples at four QC levels [Low, Medium (A), Medium (B), and High] against the response obtained from the two analytes from the post extracted plasma samples (plasma extraction was done then spiking is performed).

#### Matrix effects

The effect of human plasma components on the ionization of CAB and QUE was studied using 8 sets of the blank matrix from individual volunteers via comparing the ratio of the peak area in the presence and the absence of the matrix. The CV % should not exceed 15%.

#### Stability

The stability of CAB in the plasma matrix was evaluated using low and high QC concentrations that are examined directly after preparation and after the specific storage conditions. Five different types of stability studies were done on three replicates of each concentration as follows:

A. **Auto-sampler stability**
  The stability of extracts is studied in the auto-sampler if the auto-sampler storage conditions are different. It was done by maintaining the QC samples for 6 h directly before analysis at room temperature.
B. **Bench-top stability**
  The stability of samples under the laboratory handling conditions that are expected for the examined samples was done by keeping samples at room temperature for 24 h.
C. **Freeze–thaw stability**
  The stability of the samples after three freeze–thaw cycles was evaluated. QC samples were thawed and analyzed after being frozen for 12 h between cycles then they were thawed for 2 h at room temperature then they were frozen at − 86 °C overnight for each cycle. Freeze and thaw stability samples were assessed after each cycle.
D. **Long-term stability**
  The long-term stability was assessed over a period which is equal to or higher than the time between the date of first sample collection and the date of last sample analysis (130 days) at − 86 °C.
E. **Stock solution stability**
  Stock solution stability was assessed after 6 h at room temperature and after 8 days during short-term storage at 5 °C ± 3 °C.
  The samples were accepted in terms of stability when each QC concentration mean recovery was within ± 15 with RSD, % not exceeding 15%.

### Application for real human plasma samples

After completion of parameters of validation, the investigated method was used for the analysis of CAB amounts in the real human plasma samples of 24 healthy Egyptian volunteers (their average ages were 30 ± 10 years). The number of volunteers in a bioequivalence study should not be less than twelve as recommended by European guidelines for bioequivalence study [26]. The importance of the study, the goals, and the risks were illustrated for the volunteers after the ethics committee of Bioequivalence and stability study center (Cairo, Egypt). A single oral dose of tested product (0.5 mg CAB Film Coated tablet) and reference product (Dostinex® 0.5 mg CAB tablets) was administered to the healthy volunteers under feeding state in a cross over study design. Two tablets (1.0 mg CAB) were given as a single dose for both tested and reference products because of too low plasma concentration to be detected if one tablet only was given as a single dose (0.5 mg CAB). Human blood samples of approximately 5.00 mL were taken from volunteers and heparinized according to the proposed time intervals as follows: 0.00, 0.25, 0.5, 0.75, 1.00, 1.33, 1.67, 2.00, 2.33, 2.67, 3.00, 3.33, 3.67, 4.00, 5.00, 6.00, 8.00, 10.00, 12.00, 24.00, 48.00, and 72.00 h. The studied samples were centrifuged directly for 10 min in the presence of an anticoagulant (ethylene diamine tetra acetic acid). Then the labeled plasma samples were stored at − 86 °C till analysis. Samples preparation procedures were repeated as described before and the concentration of CAB was estimated from the constructed calibration curve.

### Pharmacokinetic (PK) parameters evaluation

Licensed Phoenix WinNonlin phoenix 64 version 8.3.2.116 software was used for pharmacokinetic (PK) calculations. Pharmacokinetic parameters of CAB were determined using the non-compartmental methods from the measured plasma concentrations. The following primary and secondary pharmacokinetic parameters were calculated. Primary parameters include maximum observed plasma concentration (C_max_) and area under the plasma concentration–time curve from zero to the last measurable concentration (AUC_0–t last_). Besides, secondary items include time to reach maximum observed plasma concentration (T_max_) and plasma concentration half-life (t_1/2_).

## Results and discussions

LC/MS/MS is a commonly used method in bioequivalence studies [27–33] for drugs detection in the plasma because of the high accuracy and sensitivity of the mass detector. Additionally, mass spectrophotometric detector is preferred over the UV and Refractive index (RI) detectors in terms of sensitivity as it measures in nano-scale levels [34, 35].

### Sample extraction and preparation

**Fig. 2:**
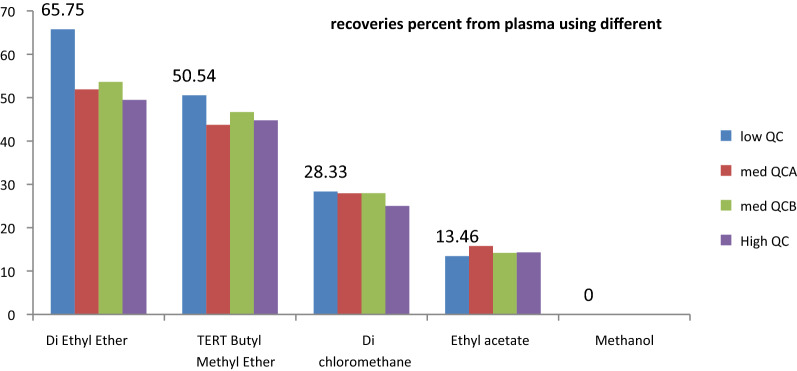
Extraction recoveries for CAB 4 different QC concentrations in human plasma using five different organic solvents

Dissimilar extracting procedures were tested to get the maximum extraction recoveries for CAB in human plasma. Protein precipitation (PP) via organic solvent was tested first; however, very poor recoveries were noticed. In contrast, liquid–liquid extraction procedures (LLE) with many organic solvents (examples were declared in 2.4.3) were tried and yielded acceptable recoveries where the best recoveries percentages were recorded in the case of diethyl ether as illustrated in Fig. 2.

### LC/MS/MS conditions

**Fig. 3:**
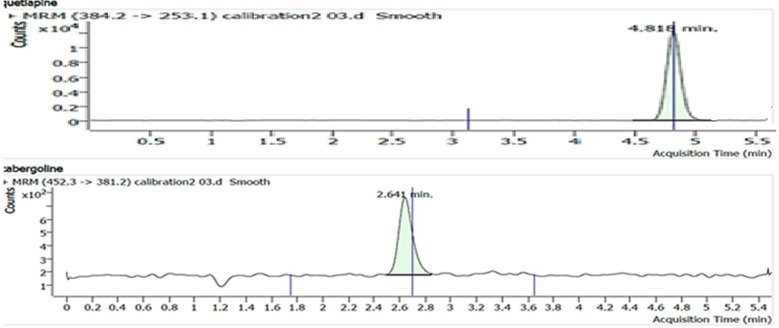
The lower chromatogram represents CAB peak in LLOQ sample and the upper chromatogram represents QUE peak at LLOQ sample

Many chromatographic parameters were carefully studied to achieve optimal resolution in short retention time, these parameters including ratio of aqueous-organic solvents, type of analytical column, and flow rate. The optimum sensitivity was fulfilled using 20 mm ammonium acetate and Methanol in the ratio (30: 70, V/V) in isocratic elution mode at a rate of 0.75 mL/min. The chromatographic separation for CAB and QUE was tested on different stationary phases e.g. Agilent eclipse plus C_8_ (4.6 × 100 mm, 3.5 µm), Agilent eclipse plus C_18_ (4.6 × 50 mm, 3.5 µm), and Agilent eclipse plus C_18_ (4.6 × 100 mm, 3.5 µm) where the best chromatogram was achieved using Agilent eclipse plus C_18_ column in terms of sensitivity and resolution values. The peaks of CAB and QUE showed acceptable symmetry and sensitivity within 5 min only as proved in Fig. 3. The separation conditions mentioned in 2.3 were applied and the optimum chromatograms for the finest separation of CAB and QUE from the matrix are illustrated in Fig. 3 and it confirms the minimum interference of the matrix with both peaks.

**Fig. 4:**
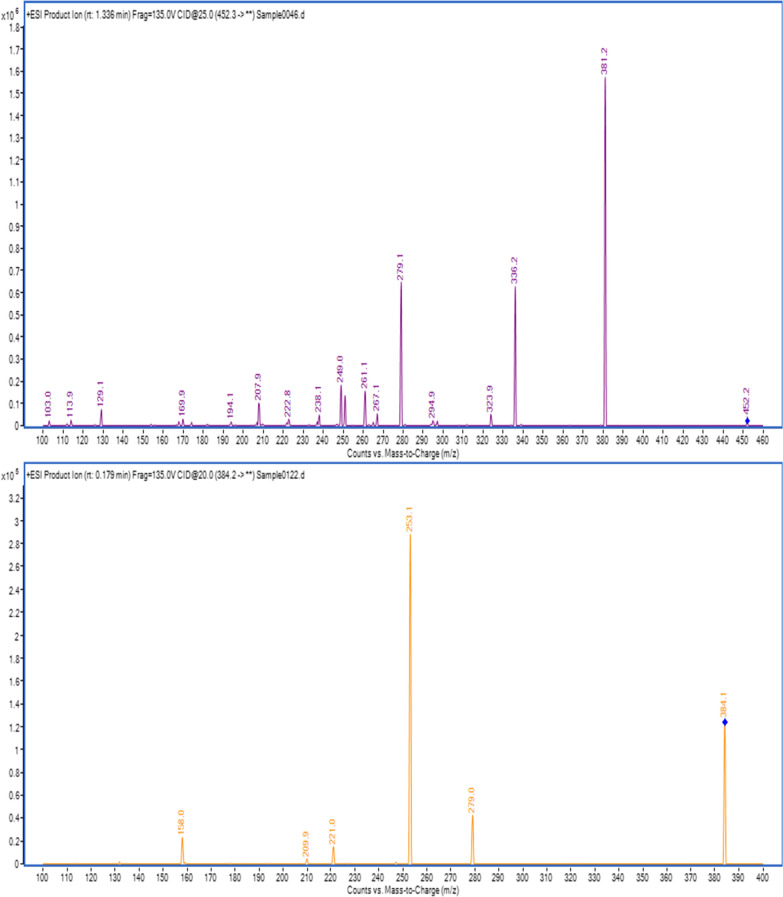
product ions scan of CAB (Analyte) and QUE (IS)

Working alcoholic solutions of CAB and QUE (100.00 ng/mL in methyl alcohol) were injected in the MS instrument to record the intact and fragment ions. Both modes of ionization (the positive and negative ion modes) were tried. The flawless intensities for the intact and fragment ions of CAB and QUE were accomplished in the positive mode as these produced ions could be protonated because of the existence of many N and O atoms. The mass spectrometer was adjusted according to the settings mentioned in 2.3. The full mass spectra were recorded from 100 to 460 m/z for CAB and from 100 to 400 m/z for QUE. The monitored [M+H]^+^ ions were m/z 452.3 → 381.2 for CAB Fig. 4 with fragmentation voltage (135 V) and Collagen Energy (25 V), while the ionization of m/z 384.2 → 253.1 for QUE Fig. 4, with fragmentation voltage (150 V) and the same Collagen Energy. The precursor ions were 452 and 384 for CAB and QUE, correspondingly. Besides, the fragment ions were recorded in the MRM mode after collision with the required energy and they were 381.2, 336.2, and 279.1 for CAB and 279.0 and 253.1 for QUE as demonstrated in Fig. 4.

### Validation of the new LC/MS/MS method

Validation protocol for FDA [26] was followed in all steps for the new LC/MS/MS method.

#### Method specificity

The recorded chromatograms (Fig. 3) for the human plasma spiked with QUE and CAB at LLOQ (2 pg/mL) three hours after taking 2 tablets having 1.00 mg of CAB confirm the method specificity where no interfering peaks for any components of the human plasma appeared in the same eluting times for CAB and QUE as well.

#### Carryover

Carryover is the appearance of an analyte peak contained in the sample of the previous sample. It was clear from the results gotten that there was no carryover effect which was well defined in the blank samples as carry over was < 20% of LLOQ and 5% of the response for QUE (IS).

#### Linearity ranges

Linearity was performed by considering the average of 6 estimations, in the range of 2.00–200.00 pg/mL for CAB. The regression equation was represented as follows:

$${\text{y}} = 1.2998{\text{x}} + 0.01706,\;{\text{r}} = 0.9978\;{\text{for}}\;{\text{CAB,}}$$

where x is the concentration for CAB in pg/mL, and y is the ratio of peak areas of CAB to QUE (IS).

Zero and Blank trials were also involved in the analysis results to confirm the absence of plasma component interference. The values of the back-calculated concentrations for the 8 points of the CAB calibration curve were within the range from 85.00 to 115.00 of the nominal concentration levels. The lower limit of quantitation (LLOQ) was 2.00 pg/mL for CAB that assures the sensitivity of the suggested method. The peak of this concentration showed a signal-to-noise ratio of 10 and RSD was less than 20%.

#### Results of accuracy and precision

Five various concentrations (LLOQ, low QC, medium QC (A), medium QC (B), and high QC) were investigated with 6 replicates for each concentration. The results illustrated in detail in Table 2 exposed that the recoveries for intra-day accuracy for day1 were ranged from 95.88 to 105.38% with a precision of 0.089–2.54% for CAB. While, the inter-day accuracy results for day3 (N = 18) were ranged from 97.63 to 101.54% with RSD, % of 0.219–5.248%. The gotten data in Table 2 assured the appropriateness of the method in terms of accuracy and precision.

**Table 2:** Accuracy and precision for the analysis of CAB in human plasma samples

	LLOQ 2 pg/mL	Low QC 6 pg/mL	Med QC (A) 20 pg/mL	Med QC (B) 60 pg/mL	High QC 160 pg/mL
**Day 1 intra-day accuracy (N = 6)**					
Equation (1/X)	Y = 1.299784 X − 0.017059 (R^2^ = 0.9978)				
STD	0.098	0.389	0.423	2.350	2.535
CV%	4.67	6.77	2.02	3.75	1.59
Accuracy%	105.38%	95.88%	104.64%	104.42%	99.71%
**Day 2 intra-day accuracy (N = 6)**					
Equation (1/X)	Y = 1.078651X − 0.030379 (R^2^ = 0.9968)				
STD	0.225	0.123	0.263	2.203	7.607
CV%	11.917	2.118	1.400	3.737	4.941
Accuracy%	94.31	97.15	93.75	98.24	96.22
**Day 3 inter-day results (N = 18)**					
Equation (1/X)	Y = 1.238189*X + 0.027894 (R^2^ = 0.9944)				
STD	0.219	0.472	1.176	3.082	5.248
CV%	11.107	8.054	5.792	5.119	3.326
Accuracy%	98.46%	97.63%	101.54%	100.35%	98.60%

*Results were excluded if they were out of the acceptance range (85–115%)

#### Efficiency of the extraction process

The efficiency of extraction procedures was evaluated via measuring 4 QC concentrations for CAB and QUE (IS) in human plasma after six repeats. The recoveries of extracted samples were compared to neat solutions of the equivalent QC levels then multiplying to factor equals 1.167 of the extraction process as 3 mL of 3.5 of organic solvent was withdrawn. The extraction recoveries for CAB were 57.71 ± 3.33 for HQC level and 76.73 ± 11.46 for LQC level, as illustrated in Table 3. The reliability and validity of extraction procedures for the new LC/MS/MS method were confirmed via results in Table 3.

**Table 3:** Results of extraction absolute recoveries and matrix effects for the analysis of CAB and QUE by the novel LC/MS/MS method in human plasma

QC levels	Extraction absolute recoveries %		Matrix effects	
	CAB	QUE (IS)	CAB	QUE (IS)
LQC	76.73 ± 11.46	58.2 ± 6.20	110.91 ± 12.43	91.46 ± 2.53
MQC(A)	60.55 ± 1.84	66.85 ± 0.39	–	–
MQC(B)	62.57 ± 6.33	65.49 ± 0.56	–	–
HQC	57.71 ± 3.33	63.27 ± 0.55	100.55 ± 4.95	108.36 ± 3.65

The extraction factor was used due to withdrawal of 3 mL form 3.5 mL of diethyl ether used in extraction

Mean percentage of absolute recoveries and RSDs were estimated for six peak areas of various plasma samples

Absolute recoveries calculations were calculated by dividing mean area of extracted samples over mean area of un-extracted samples then multiplied by the extraction factor 1.167 and 100 (Ex. recovery of analyte at low concentration = (12,371.5/18815.5) * 1.167 * 100 = 76.73%)

#### Investigating of matrix effect

Matrix human plasma components have a significant influence on drug ionization. The outcomes of CAB and QUE recoveries illustrated in Table 3 reveal that the matrix constituents have no effects on the ionization of CAB and QUE (IS).

#### Investigation of CAB stability

Recommendations of FDA guidelines for bioanalytical method validation were followed for assessment of CAB stability during handling, extracting and analysis. Results of stability tests were collectively displayed in Additional file 1: Table S1, where 2 different concentrations (low and high QC samples) were estimated in different conditions. CAB was stable for 40 h during storage at room temperature and after 8 days during short-term storage at 5 °C ± 3 °C. Besides, CAB was stable after three cycles of freeze and thaw at − 70 °C where the recoveries at third cycle were 100.39 ± 8.65 for the low QC sample and 92.97 ± 2.67 for the high QC sample. Furthermore, CAB was stable for nearly 130 days of long-term stability at − 86° ± 15 °C.

### Application of the new LC/MS/MS method for determination of real plasma samples for Egyptian volunteers

**Fig. 5:**
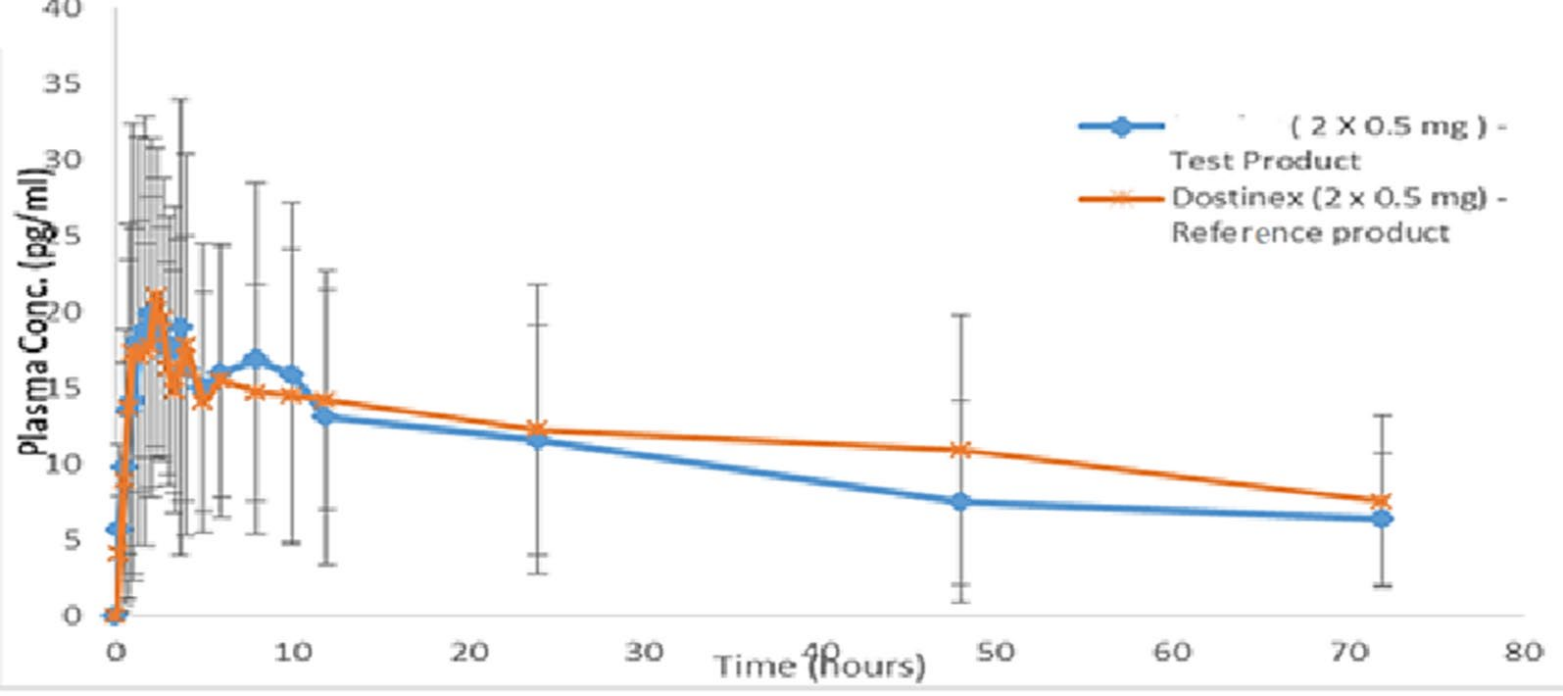
Mean plasma concentration versus time profile with standard deviation bars after a single dose of Test product (2 × 0.5 mg) film coated tablets and Dostinex (2 × 0.5 mg) tablets (reference product)

The novel method was fruitfully used for the analysis of CAB in human plasma for twenty-four healthy volunteers in Cairo (capital of Egypt). The two products [Dostinex: reference and test product] were administrated as a single dose of two tablets orally under feeding state. Besides, plasma concentrations of CAB were determined at various time intervals from 0 to 72 h as illustrated in Additional file 1: Table S2 and Fig. 5. It is clear from Fig. 5 that there were no differences between plasma concentration curves for the reference (Dostinex) and test products.

### Results of pharmacokinetic parameters

The results of pharmacokinetic parameters are summarized in Table 4 as follows; C_max_ were 29.37 ± 7.5, and 30.20 ± 3.40 pg/mL; T_max_ were 2.17, and 2.33 h; AUC_0–t_ were 734.38 ± 174.66 and 755.62 ± 143.29 pg·h/mL for test product and Dostinex, respectively. The median values of T_max_ (h), were 2.17 h and 2.33 h for test product and Dostinex, correspondingly as displayed in Table 4. According to the attained results (C_max_, AUC_0–72_, T_max_, t_½_). There was no significant difference between test product (0.5 mg film-coated tablets) and Dostinex 0.5 mg tablets (reference product). Test tablets are considered bioequivalent to Dostinex tablets and could be approved as a good substitute for the generic product.

**Table 4:** Pharmacokinetics parameters of Cabergoline after a single dose of test product (2 × 0.5 mg) film coated tablets and Dostinex (2 × 0.5 mg) tablets (reference product)

	Items	C_max_ (pg/mL)	T_max_ (h)	AUC_0-t_ (pg·h/mL)	K_el_ (1/h)	t½ (h)
Test product (2 × 0.5 mg) tablets	Mean	29.37	2.17	734.38	0.017	131.221
	CV	± 7.5	± 0.2	± 174.66	± 0.012	± 33.506
Reference product (2 × 0.5 mg) tablets	Mean	30.20	2.33	755.62	0.02	80.99
	CV	± 3.40	± 0.25	± 143.29	0.02	± 13.46

### Estimation for the greenness of the proposed LC/MS/MS method

AGREE approach is simple and reliable for the assessment of eco-friendly characters of the analytical method [19, 21, 36–43]. The final greenness numerical value was 0.63 with a relatively pale green color inside the pictogram as demonstrated in Additional file 1: Fig. S1 which illustrates the eco friendlessness characters for the novel method. The use of the acetonitrile-free mobile phase is one of the merits of the method because of its known hazards to the environment. The most hazardous red subsections in the pictogram are sectors 3 and 9. Sector 3 denotes off-line sampling while sector 9 refers to the used intensive energy instrument like LC/MS. The analysis of CAB and QUE in 5.5 min only which permits the analysis of a large number of samples per hour is understood from full green sector 8 in the pictogram.

### Advantages of the novel LC/MS/MS method over the reported methods

Generally, up to our recent knowledge, CAB was analyzed formerly in plasma via LC/MS/MS by two methods [17, 18]. The first LC/MS/MS method [17] which was developed by Allievi and Dostert involved using an expensive Deutrated internal standard which is not available in most QC labs. Moreover, this method was not tried for CAB detection in real volunteers’ human plasma for pharmacokinetics researches as illustrated in Table 5. While the second LC/MS/MS [18] reported by Igarashi et al. was applied for the simultaneous analysis of CAB and L-dopa in human plasma which is suitable for concurrent administration of the two drugs in case of parkinsonism only. Besides, this method was used a large volume of acetonitrile up to 80% in gradient mode which is not favored in terms of green analytical chemistry. Accordingly, our novel method is considered the first one which achieved validated LC–MS/MS trial for CAB analysis in presence of cheap QUE (IS) in human plasma with an adequate degree of accuracy, reliability, and sensitivity. Moreover, LOD and LOQ of the method were found to be 0.5 and 1.6 pg/mL respectively. Furthermore, the new method was characterized by using acetonitrile-free extracting solvent and mobile phase that assures its priority in terms of method greenness. No adjustment of pH for the mobile phase is required and using simple isocratic elusion mode are merits of the new methods too. The sensitivity of the three methods is almost the same as all of them measured CAB in pictogram scale, linearity ranges are recorded in Table 5. Regarding total run time, our new method has the shortest time about 5.5 min which means less solvent consuming, more economic, and safer for the environment.

**Table 5:** Comparison between the different reported LC–MS/MS methods for CAB in plasma [17, 18] and the new suggested one

Analyte/application	Volume of human plasma	Extraction solvent composition/volume	Blood Volume	Internal standard	Components of Mobile system	Flow rate (mL*/*min)	Retention time/total run time	Stationary phase	Linear range of CAB (pg/mL)	The AGREE score	References
CAB alone/applied for pure CAB only in plasma samples but not for human volunteers	150 µL	A mixture of methylene chloride/isooctane, 2:3/2.5 mL	1 mL	Deutrated C14 labeled CAB	Aqueous solution of ammonium formate (10 mM, pH 3): acetonitrile (70/30, v/v)	0.5	4.26 min/4.27 min	A µ-Bondapak C18 (3.9 × 150 mm, 10 μm)	1.86–124.0	0.36	Allievi and Dostert [21]
CAB and L-dopa/for schizophrenic patients	100 µL	Acetonitrile, 20 mM ammonium formate liquid (90:10, v/v)/50 µL	Not mentioned	Deutrated C14 CAB	Gradient elution mode started with 20; 80 V, V acetonitrile; water (containing 20 mM ammonium formate) for 3 min	0.2	7.4 min/2.5 min	RP-COSMOSIL C8-MS column (2 mm × 150 mm, 5-mm diameter, Kyoto, Japan)	5–250	0.47	Igarashi et al. [22]
CAB alone/in pharmaceutical product analysis and pharmacokinetics studies	500 µL	Diethyl ether/3 mL	5 mL	Quetiapine	Isocratic mode, methanol: 20 mm ammonium acetate (70: 30, V/V)	0.75	2.6 min/5.5 min	Agilent eclipse reversed-phase C_18_ (3.5 µm, 4.6 × 100 mm) column	2–200	0.63	Our new method

However, the other two methods have the advantage of using labeled isotope IS for CAB which is much related chemically to the drug. Unfortunately, its price is too costly and makes the repeatability of the method unsuitable for routine QC analysis. In our novel method, Quetiapine (QUE) was selected as a structure correlated compound that is expected to show the same behavior as the CAB in both extraction and separation processes.

### Commendations from this research paper

Besides, test product tablets are considered bioequivalent to Dostinex® tablets and could be approved as a good substitute generic product for the brand product of Pfizer.

### Future research and limitation of the study

Based on the results, the QUE and CAB have dissimilar behaviors in chromatographic separation since the elution times are different by over 2 min. The authors admit that the choice of internal standard is a drawback for this study; although the outcomes meet the acceptance criteria. The novel method could be used for testing other generic formulations available in Egyptian pharmacies e.g. Cabergamoun, Marvigoline, Elona, and Nostifix. Moreover, repeating the pharmacokinetic study for CAB on human volunteers with different nationalities is strongly recommended to investigate the effect of genetic variations on pharmacokinetics parameters for the drug.

## Conclusion

A novel, rapid, green, and valid LC–MS/MS method was established for the analysis of CAB in human plasma for healthy Egyptian volunteers. The method was completely validated according to the protocol of FDA Guidance for industry: Bio-analytical Method Validation. The accuracy, selectivity, and precision of the novel method were tested successfully with satisfying results. Solvent extraction technique using diethyl ether yields the highest recoveries for CAB from human plasma. The merits of the method over the previous published methods are low cost; availability of cheap internal standard; short analysis time; use of acetonitrile free solvents mobile phase; applicability for CAB analysis in real human plasma samples.

Finally, regarding the tested product evaluation, the generic formulation of test product (0.5 mg tablets) was considered to be bioequivalent to the reference product Dostinex 0.5 mg tablets under study conditions, and test product satisfies the requirements for the Egyptian authority**.**

## Supplementary Information

**Additional file 1: Figure S1.** AGREE approach for estimation of new LC/MS/MS method greenness for CAB. **Figure S2.** AGREE approach for estimation of old LC/MS method greenness for CAB by Allievi and Dostert, in 1998. **Figure S3.** AGREE approach for estimation of old LC/MS/MS method greenness for CAB by Igarashi et al. in 2003. **Table S1.** The details for Recovery calculations of Cabergoline. **Table S2.** The details for Recovery calculations of QUE (IS). **Table S3.** Matrix effect of Cabergoline. **Table S4.** Outcomes of stability studies in different environments for the CAB QC samples in human plasma samples. **Table S5.** Mean Plasma concentrations of CAB versus time after a single dose administration of 2 tablets of test product (0.5 mg Film Coated tablet) and 2 tablets of Dostinex 0.5 mg tablets (reference product).

## Data Availability

We declare that all data include information that support the results reported in the article is available upon request.
