# Supplementary material for: A validated LC–MS/MS method for analysis of Cabergoline in human plasma with its implementation in a bioequivalent study: investigation of method greenness
Source: BMC Chem. 2022 Sep 24;16(1):71. doi: 10.1186/s13065-022-00862-6 (PMC9509579; doi:10.1186/s13065-022-00862-6)
Supplement: Supplementary file 1 — Additional file 1: Figure S1. AGREE approach for estimation of new LC/MS/MS method greenness for CAB. Figure S2. AGREE approach for estimation of old LC/MS method greenness for CAB by Allievi and Dostert, in 1998. Figure S3. AGREE approach for estimation of old LC/MS/MS method greenness for CAB by Igarashi et al. in 2003. Table S1. The details for Recovery calculations of Cabergoline. Table S2. The details for Recovery calculations of QUE (IS). Table S3. Matrix effect of Cabergoline. Table S4. Outcomes of stability studies in different environments for the CAB QC samples in human plasma samples. Table S5. Mean Plasma concentrations of CAB versus time after a single dose administration of 2 tablets of test product (0.5 mg Film Coated tablet) and 2 tablets of Dostinex 0.5 mg tablets (reference product). [file 13065_2022_862_MOESM1_ESM.docx]

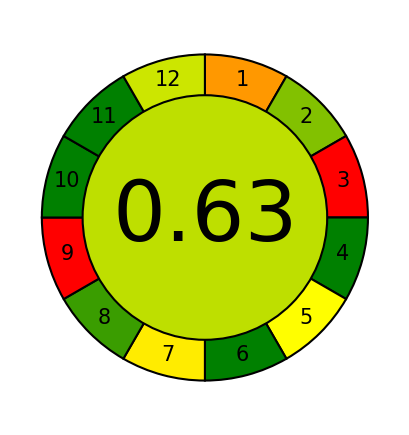


**Figure S1:** AGREE approach for estimation of new LC/MS/MS method greenness for CAB


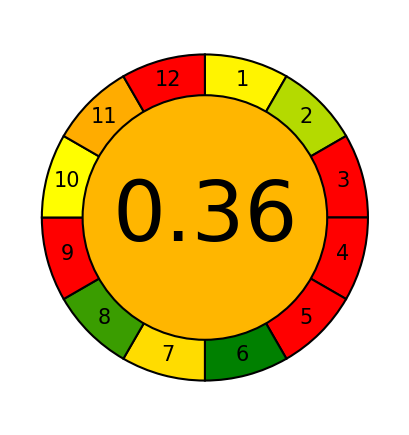


**Figure S2:** AGREE approach for estimation of old LC/MS method greenness for CAB by Allievi and Dostert, in 1998.


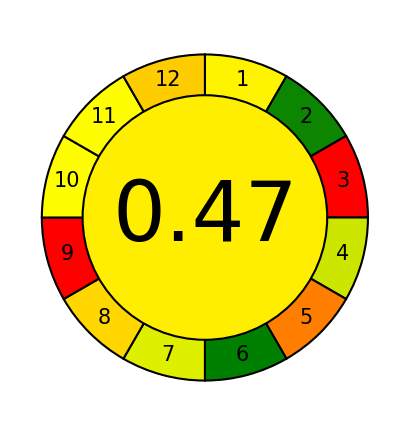


**Figure S3:** AGREE approach for estimation of old LC/MS/MS method greenness for CAB by Igarashi et al in 2003.

**Table S1:** The details for Recovery calculations of Cabergoline

| **SN** | **Low QC (N=6)** | | **Med QC (A) (N=6)** | | **Med QC (B) (N=6)** | | **High QC (N=6)** | |
| --- | --- | --- | --- | --- | --- | --- | --- | --- |
|  | **Un-extracted**  **(peak area)** | **Extracted**  **(peak area)** | **Un-extracted**  **(peak area)** | **Extracted**  **(peak area)** | **Un-extracted**  **(peak area)** | **Extracted**  **(peak area)** | **Un-extracted**  **(peak area)** | **Extracted**  **(peak area)** |
| **1** | 18208 | 11126 | 31285 | 15587 | 93150 | 54277 | 268691 | 137346 |
| **2** | 19429 | 12602 | 30411 | 16234 | 92787 | 52130 | 273114 | 143289 |
| **3** | 19651 | 13493 | 30105 | 15772 | 94526 | 49922 | 277953 | 138733 |
| **4** | 17230 | 14502 | 30978 | 15917 | 94008 | 49626 | 280825 | 135912 |
| **5** | 19425 | 11525 | 29998 | 15379 | 94231 | 44790 | 279174 | 129389 |
| **6** | 18950 | 10981 | 29660 | 15788 | 94027 | 51042 | 278189 | 135424 |
| **Mean** | **18815.5** | **12371.5** | **30406.17** | **15779.5** | **93788.1667** | **50297.83** | **276324.3** | **136682.2** |
| **STD** | **932.4044** | **1417.833** | **618.6163** | **290.8819** | **671.633655** | **3183.496** | **4540.488** | **4553.312** |
| **CV%** | **4.96** | **11.46** | **2.03** | **1.84** | **0.72** | **6.33** | **1.64** | **3.33** |
| **Absolute Recovery** | **76.71** | | **60.55** | | **62.57** | | **57.71** | |

**Table S2:** The details for Recovery calculations of QUE (IS)

| **SN** | **Low QC (N=6)** | | **Med QC (A) (N=6)** | | **Med QC (B) (N=6)** | | **High QC (N=6)** | |
| --- | --- | --- | --- | --- | --- | --- | --- | --- |
|  | **Un-extracted**  **(peak area)** | **Extracted**  **(peak area)** | **Un-extracted**  **(peak area)** | **Extracted**  **(peak area)** | **Un-extracted**  **(peak area)** | **Extracted**  **(peak area)** | **Un-extracted**  **(peak area)** | **Extracted**  **(peak area)** |
| **1** | 159984 | 68669 | 163918 | 93942 | 187264 | 107301 | 173958 | 94885 |
| **2** | 158833 | 70114 | 165586 | 94694 | 189956 | 106691 | 173758 | 94475 |
| **3** | 158029 | 69210 | 164162 | 94874 | 190238 | 106068 | 172641 | 94178 |
| **4** | 130890 | 71094 | 165046 | 94109 | 188582 | 105582 | 173168 | 93862 |
| **5** | 134009 | 77641 | 163611 | 94256 | 190710 | 106661 | 173782 | 93384 |
| **6** | 131604 | 78973 | 165453 | 94153 | 190862 | 106308 | 174441 | 94214 |
| **Mean** | **145558.167** | **72616.83333** | **164629.333** | **94338** | **189602** | **106435.167** | **173624.667** | **94166.3333** |
| **STD** | **14718.048** | **4503.73629** | **840.07706** | **364.482647** | **1403.68942** | **590.761345** | **631.852409** | **513.581412** |
| **CV%** | **10.11** | **6.20** | **0.51** | **0.39** | **0.74** | **0.56** | **0.36** | **0.55** |
| **Absolute Recovery** | **58.2** | | **66.85** | | **65.49** | | **63.27** | |

**Table S3:** Matrix effect of Cabergoline

| **SN** | **Low QC (N=6)** | | | | | | |
| --- | --- | --- | --- | --- | --- | --- | --- |
|  | **Analyte Unextracted**  **(peak area)** | **Analyte Extracted**  **(peak area)** | **Analyte Matrix factor** | **IS Unextracted**  **(peak area)** | **IS Extracted**  **(peak area)** | **IS Matrix factor** | **Normalized factor** |
| **1** | 11357 | 12191 | 0.98 | 165487 | 154909 | 0.94 | 1.05 |
| **2** | 11839 | 12175 | 0.98 | 170036 | 153628 | 0.93 | 1.05 |
| **3** | 11697 | 14735 | 1.19 | 166980 | 153295 | 0.93 | 1.28 |
| **4** | 11464 | 16646 | 1.34 | 163158 | 150765 | 0.91 | 1.47 |
| **5** | 12289 | 13179 | 1.06 | 163189 | 148248 | 0.90 | 1.18 |
| **6** | 15778 | 13618 | 1.10 | 161046 | 144858 | 0.88 | 1.25 |
| **Mean** | **12404.000** | **13757.333** | **1.109** | **164982.667** | **150950.500** | **0.91** | **1.21** |
| **STD** | **1684.992** | **1709.461** | **0.138** | **3219.437** | **3815.544** | **0.023** | **0.158** |
| **CV%** | **13.58** | **12.43** | **12.43** | **1.95** | **2.53** | **2.53** | **13.02** |
| **SN** | **High QC (N=6)** | | | | | | |
|  | **Analyte Unextracted**  **(peak area)** | **Analyte Extracted**  **(peak area)** | **Analyte Matrix factor** | **IS Unextracted**  **(peak area)** | **IS Extracted**  **(peak area)** | **IS Matrix factor** | **Normalized factor** |
| **1** | 292034 | 267612 | 0.91 | 152907 | 154173 | 1.03 | 0.88 |
| **2** | 295127 | 302736 | 1.03 | 151554 | 156476 | 1.05 | 0.98 |
| **3** | 295131 | 307843 | 1.04 | 149079 | 159808 | 1.07 | 0.97 |
| **4** | 296144 | 304818 | 1.03 | 146615 | 163724 | 1.10 | 0.94 |
| **5** | 295018 | 298696 | 1.01 | 147413 | 167110 | 1.12 | 0.90 |
| **6** | 295440 | 296982 | 1.01 | 147290 | 168361 | 1.13 | 0.89 |
| **Mean** | **294815.667** | **296447.833** | **1.006** | **149143.000** | **161608.667** | **1.08** | **0.928** |
| **STD** | **1423.322** | **14671.710** | **0.050** | **2561.328** | **5748.202** | **0.039** | **0.043** |
| **CV%** | **0.48** | **4.95** | **4.95** | **1.72** | **3.56** | **3.56** | **4.63** |

**Table S4.** Outcomes of stability studies in different environments for the CAB QC samples in human plasma samples.

| **Stability Environments** | **CAB** | |
| --- | --- | --- |
|  | **LQC (n=3)**  **Recovery %**  **± RSD,%** | **HQC (n=3)**  **Recovery % ± RSD,%** |
| **Auto-sampler stability:** | 102.56 ± 0.29  (after 48 hours) | 89.63 ± 0.31  ( after 72 hours) |
| **Short term stability (N=3)** | 101.32± 7.41  (after 40 hours) | 87.75 ± 1.57  (after 40 hours) |
| **Dry extract stability (N=3)** | 102.14± 7.47  (after first cycle) | 94.63± 11.76  (after first cycle) |
| **Freeze and thaw stability (1^st^ cycle) (N=3)** | 98.39 ± 10.32 | 86.32 ± 0.62 |
| **Freeze and thaw stability (2^nd^ cycle) (N=3)** | 106.83 ± 7.47 | 101.66 ± 9.08 |
| **Freeze and thaw stability (3^rd^ cycle) (N=3)** | 100.39 ± 8.65 | 92.97 ± 2.67 |
| **Long term stability (N=3)** | 97.43 ± 9.82 | 97.96 ± 0.77 |
| **Stock solution stability after 6 h. at room temperature (N=6)** | 97.46 ± 2.79 | 99.99 ± 0.36 |
| **Stock solution stability 7 days at (2-8 ºC) (N=6)** | 98.55 ± 1.83 | 103.41 ± 1.07 |

**Table S5:** Mean Plasma concentrations of CAB versus time after a single dose administration of 2 tablets of test product (0.5 mg Film Coated tablet) and 2 tablets of Dostinex 0.5mg tablets (reference product)

| Time Interval | Test product ( 2 × 0.5 mg ) | | Reference product (2 × 0.5 mg) | |
| --- | --- | --- | --- | --- |
|  | **Mean** | **SD** | **Mean** | **SD** |
| Pre dose | 0.00 | 0.00 | 0.00 | 0.00 |
| 0.25 | 5.721 | 5.528 | 4.084 | 3.813 |
| 0.5 | 9.776 | 8.991 | 8.936 | 7.8 |
| 0.75 | 13.498 | 12.33 | 13.733 | 9.64 |
| 1 | 14.151 | 11.327 | 17.333 | 15.015 |
| 1.33 | 18.041 | 13.457 | 17.059 | 8.894 |
| 1.67 | 18.753 | 14.129 | 17.424 | 7.078 |
| 2 | 19.912 | 11.434 | 17.691 | 9.875 |
| 2.33 | 19.586 | 9.2 | 21.00 | 9.808 |
| 2.67 | 18.076 | 7.548 | 19.498 | 9.257 |
| 3 | 17.438 | 8.842 | 16.261 | 7.014 |
| 3.33 | 17.462 | 9.399 | 14.764 | 7.967 |
| 3.67 | 19.03 | 14.991 | 16.175 | 8.627 |
| 4 | 16.305 | 8.681 | 17.824 | 12.55 |
| 5 | 14.967 | 9.523 | 14.085 | 7.219 |
| 6 | 16.041 | 8.225 | 15.499 | 8.998 |
| 8 | 16.94 | 11.596 | 14.73 | 7.103 |
| 10 | 15.941 | 11.227 | 14.514 | 9.588 |
| 12 | 13.045 | 9.634 | 14.214 | 7.21 |
| 24 | 11.548 | 7.586 | 12.29 | 9.499 |
| 48 | 7.531 | 6.688 | 10.892 | 8.906 |
| 72 | 6.38 | 4.341 | 7.55 | 4.61 |

***Mean concentration of data of 24 human volunteers**
